# A Spray-On Carbon Nanotube Artificial Neuron Strain Sensor for Composite Structural Health Monitoring

**DOI:** 10.3390/s16081171

**Published:** 2016-07-26

**Authors:** Gyeongrak Choi, Jong Won Lee, Ju Young Cha, Young-Ju Kim, Yeon-Sun Choi, Mark J. Schulz, Chang Kwon Moon, Kwon Tack Lim, Sung Yong Kim, Inpil Kang

**Affiliations:** 1 Manufacturing Automation R & BD Group, Korea Institute of Industrial Technology, Chonan 143-701, Korea; grchoi@kitech.re.kr; 2 Department of Architectural Engineering, Namseoul University, Cheonan 331-707, Korea; jwlee@nsu.ac.kr; 3 Department of Medical Device, Korea Institute of Machinery & Materials, Daegu 711-880, Korea; jycha@kimm.re.kr; 4 Mineral Resources Research Division, Korea Institute of Geoscience and Mineral Resources, Daejeon 305-350, Korea; kyjp7272@kigam.re.kr; 5 School of Mechanical Engineering, Sungkyunkwan University, Suwon 440-746, Korea; yschoi@skku.ac.kr; 6 Smart Structures and Bio-Nanotechnology Laboratory, University of Cincinnati, Cincinnati, OH 45221, USA; Mark.J.Schulz@uc.edu; 7 Graduate School of Pukyong National University, Busan 608-739, Korea; moonck@pknu.ac.kr (C.K.M.); ktlim@pknu.ac.kr (K.T.L.); ksy1357@pukyong.ac.kr (S.Y.K.)

**Keywords:** carbon nanotube, artificial neuron, strain sensor, composites, structural health monitoring, piezoresistivity, damage detection

## Abstract

We present a nanocomposite strain sensor (NCSS) to develop a novel structural health monitoring (SHM) sensor that can be easily installed in a composite structure. An NCSS made of a multi-walled carbon nanotubes (MWCNT)/epoxy composite was installed on a target structure with facile processing. We attempted to evaluate the NCSS sensing characteristics and benchmark compared to those of a conventional foil strain gauge. The response of the NCSS was fairly good and the result was nearly identical to the strain gauge. A neuron, which is a biomimetic long continuous NCSS, was also developed, and its vibration response was investigated for structural damage detection of a composite cantilever. The vibration response for damage detection was measured by tracking the first natural frequency, which demonstrated good result that matched the finite element (FE) analysis.

## 1. Introduction

Composites are becoming crucial structural materials in high technology industries. Composites offer excellent strength/stiffness-to-weight, resistance to corrosion and chemical attack, good electrical behavior, and typically provide sustainable and low maintenance. Due to such advantages of composites, the Boeing 787 designed carbon fiber reinforced composite airframe and primary structure saves 20% more fuel than conventionally similar airplanes [1]. Despite such great merits, composites consisting of multiple laminar layers are inherently prone to failure owing to delamination of the multiple laminar layers, moisture absorption, and low speed impact. Structural applications of composites are still more challenging and less predictable than metals. 

More sophisticated structural health monitoring (SHM) and nondestructive inspection (NDI) techniques are required to detect defects of composites than metals. Traditional NDI of composites include visual inspection, automated tap tests, ultrasonic inspection, radiography, and thermography. To improve the efficiency of the visual inspection conducted with the naked eye, some techniques have been developed such as impact sensitive coatings, liquid penetrants, and magnetic particles. The vibration analysis in terms of natural frequency change is reported as an effective method compared to ultrasound transmission techniques for filament wound carbon fiber reinforced plastic (CFRP) tubes [2]. Some techniques use a bulky transducing system and need to eliminate individual components for testing, which have a high cost and are labor intensive. The inspection cost is more than one-third of acquiring and operating the structure [3]. Therefore, a more economical and effective way is required. 

A promising sensor should be light, small, and easily installed in a structure with high sensitivity to cover a large target area for an SHM. A sensor made of carbon nanotubes (CNTs) might meet such demands due to their unique properties [4,5]. CNTs can be used to form smart materials and possibly produce various kinds of sensors for composites. CNTs have been studied to develop novel sensors due to their promising structural and electrical characteristics [6,7,8,9,10]. For applying CNTs as strain sensors for engineering purposes, CNTs can be fabricated as a smart nanocomposite through a composite process [11,12,13,14,15]. In addition, a liquid type CNT sensor can also be obtained from a composite process and may be useful as a novel smart paint sensor for SHM. Smart paint has the advantage of being able to produce complex shapes of a target structure with a light sensor equipment load [16,17,18]. 

However, the CNT strain sensor is still in early development for SHM. The CNT strain sensing performance by means of voltage responses need to be systematically assessed for structural deterioration monitoring under various loading conditions. Therefore, we report the multi-walled carbon nanotube (MWCNT) composite strain sensing properties under static and dynamic loading conditions and experimentally studied the composite to develop a neuron as a continuous strain sensor. The long continuous strain sensor was named a neuron [19]. Large structures, such as bridges and buildings, need to be monitored over long spans, and the multi-dimensional strain in the structure must be measured. In large structural applications, it is difficult to install a sufficient number of conventional strain gauges because they require a large amount of wiring and electronics. However, a neuron can cover a large area and detect the strain along it due to its continuity. A CNT neuron can measure strains occurring along its length and it can be easily installed on large structures using a spray-on technique [12]. For engineering applications, it is necessary to develop more economic materials for the sensors and to perform systematic studies of the sensing characteristics. Since a displacement transducer has the advantage of monitoring a structure under large bending deflections with low frequency vibration [20], we characterized the strain sensor using the frequency responses. Structural vibration based health monitoring has been used to detect damage in a structure through altered dynamic characteristics and the change is characterized by changes in the natural frequency [21,22]. The vibration response of the neuron was investigated with experimental and finite element (FE) analysis for structural damage detection of a composite cantilever by tracking the first natural frequency.

## 2. Fabrication of the Neuron

The artificial neuron was fabricated with MWCNT and epoxy. Epoxy (Kukdo-chemical Co., Seoul, Korea, YD-128) was used as a matrix for the nanocomposite because it shows excellent mechanical strength and is easily fabricated as a composite. Commercially-obtained MWCNTs (Hanwha Chemical Co., Seoul, Korea, CM-250) were used for the nanocomposite filler. Though single-walled carbon nanotubes (SWCNTs) have excellent electromechanical properties, because of the high cost and difficult incorporation into polymers at high loading, this study used an MWCNT composite to develop a continuous strain sensor. The piezoresistive properties of MWCNT have been greatly improved due to the successful development of the synthesis process. The base MWCNT solution was prepared using a similar process to the one described in our previous study, and the piezoresistive characteristics of both PMMA and epoxy composites are nearly similar [6].

**Figure 1:**
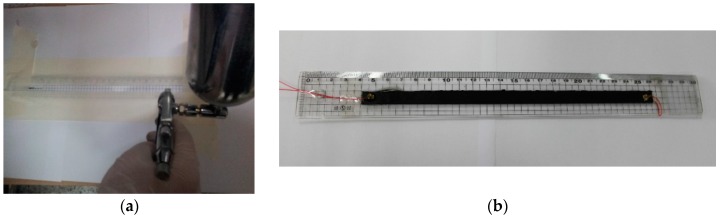
Fabrication of the multi-walled carbon nanotube (MWCNT)/epoxy neuron with spray: (**a**) spray on a patterned bar; (**b**) fabricated neuron on a plastic ruler (3 wt. % MWCNT, 220 mm × 10 mm × 0.33 mm, R = 3.07 kΩ).

The mixed MWCNT solution was sprayed with an airbrush on a patterned surface of a structure as shown in Figure 1a. The sprayed-on neuron was cured at room temperature until completely dry. The spray process was repeated several times to obtain a uniform thin layer. After the curing process, the electrical wires were connected using silver conducting epoxy (Cleantech, Ansan, Korea, Elcoat P-100) to reduce the contact resistance. An epoxy layer or another polymer layer can be optionally coated on the neuron layer to protect the neuron from contamination and external damage. Figure 1b shows the neuron on a plastic beam. Patterning was required to install the neuron on a structure with controlled sensor dimensions. The length and thickness affect the resistance of the neuron that is related to the sensitivity of the neuron. If the sensor electrode of the nanocomposite strain sensor (NCSS) is wider, the strain response of the sensor might be mixed with axial and transverse deformation. In order to read the proper strain information, the pattering of the sensor electrode should be studied. The major advantage of the spray-on neuron as a strain sensor was ease of use on complex surface shapes, including welded parts, where stress concentrations can occur and are difficult to measure using conventional strain gauges.

**Figure 2:**
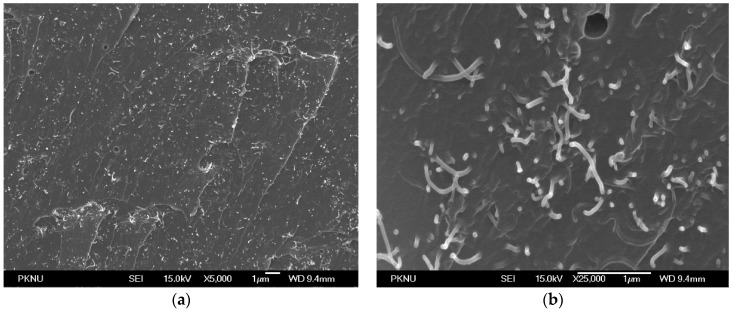
FE-SEM images of sprayed MWCNT/epoxy (3 wt. %) samples: (**a**) 5000× and (**b**) 25,000×.

Figure 2 shows the FE-SEM images of the fractured side surface of an MWCNT/epoxy composite fabricated using the same dispersion process that was used to form the neuron. Good dispersion of MWCNTs in the epoxy composite was achieved using this fabrication process and the electrically conducting percolation level was approximately 0.1 wt. %, which is normal compared to the literature [22].

## 3. Characteristics of the MWCNT/Epoxy Strain Sensor

### 3.1. Piezoresistive Characteristics of the MWCNT/Epoxy Composites

**Figure 3:**
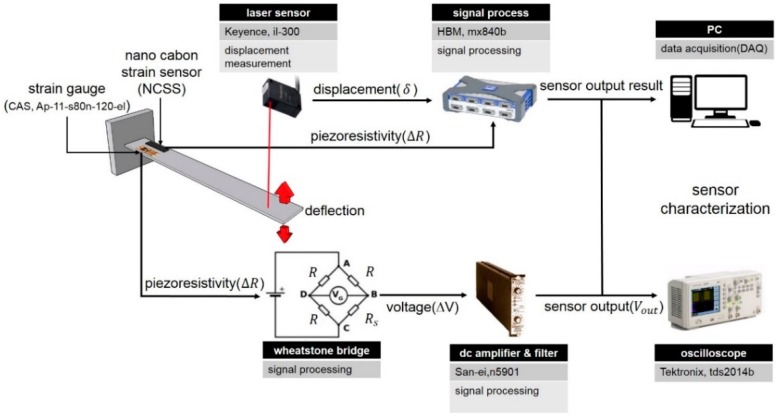
Schematic illustration of the strain measurement.

The piezoresistive characteristics of the nanocomposites were investigated to determine the variations of electrical properties under structural deformations. The film type specimens were fabricated to investigate strain sensing properties under static and dynamic loading conditions and to calibrate neurons. The short length specimens were fabricated in the same way as the nano-spray process, with the change in CNT wt. % fraction set as 0.7, 2, 3, and 10. Specimens were installed on a cantilever and connected to wires with silver conductive epoxy on the surface to build the electrodes of the NCSS. The resistance changes of the NCSS electrodes were measured with a multi-meter (Yokogawa, Tokyo, Japan, 732) and the cantilever beam deflection was measured using a laser displacement sensor (Keyence, Osaka, Japan, il-300) as shown in Figure 3.

The measured displacements were converted to strains by cantilever beam theory, and the resistance changes were normalized (*R_N_*) with Equation (1):

(1) $$R_{N}=\frac{R_{s}-R_{0}}{R_{0}}$$

**Figure 4:**
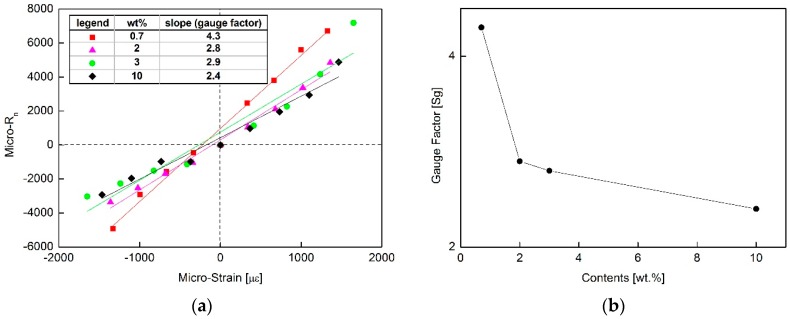
Piezoresistive characteristics of MWCNT/epoxy composites for strain sensors: (**a**) experimental strain model and (**b**) gauge factor w.r.t MWCNT wt. %.

*R*_0_ is the resistance without any displacement and *R_s_* is the measured sensor resistance when the beam is strained. Figure 4 shows the experimental strain model of the MWCNT/epoxy NCSS from the results of the above conversions. As shown in Figure 4a, the MWCNT/epoxy composites showed excellent linearity under compression and expansion loads. The slope represents the sensitivity (*S_g_*) of the fabricated NCSS and corresponds to the gauge factor of the sensor determined by Equation (2):

(2) $$S_{g}=\frac{\Delta R_{N}}{\Delta \varepsilon_{a}}$$

Here, *ε_a_* is the axial strain due to the beam deflection. To investigate the linearity and gauge factor of the MWCNT piezoresistivity, the relationship between the content ratio of MWCNT and the gauge factor of the strain sensors was investigated as shown in Figure 4b.

The piezoresistivity shows fairly linear bidirectional strain response over various contents of NCSS. The piezoresistive mechanism of CNT composites strongly relate to electrical contact resistance change of the fillers in the matrix and tunneling effects of the fillers [6,23]. The resistance change in case of lower MWCNT contents is much larger than higher contents of MWCNT composites and that makes higher sensitivity of piezoresistivity. The abundant electrical conductive networks in higher content composites may hamper the piezoresistive changes. As shown in Figure 4b, the piezoresistive sensitivity was affected by the filler concentration and the composites near the percolation threshold displayed higher sensitivity than composites at higher concentrations. To design the sensor sensitivity, the proper content point at the curve knee point can be selected and the optimized sensitivity considering the weight of MWCNT can be achieved at 2–3 wt. %.

### 3.2. Strain Sensing Characteristics of the MWCNT/Epoxy NCSS

To benchmark to a conventional strain gauge, a foil strain gauge (CAS, Yangju, Korea, AP-11 S30N-120-EC) was installed with the NCSS on the same cantilever, as illustrated in Figure 3. The NCSS should be characterized by means of its voltage responses to compare its performances with the commercial sensor, and we utilized traditional signal processing for the sensors.

Table 1 shows the specification of the foil strain gauge and the NCSS. The experiment was processed using 3 wt. % NCSS, which shows a similar gauge factor region to the foil strain gauge.

**Figure 5:**
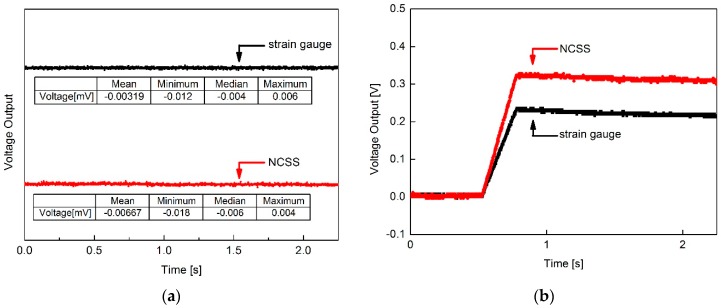
Response profiles of the MWCNT/epoxy nanocomposite strain sensor (NCSS) and strain gauge: (**a**) NCSS voltage output without a load; (**b**) NCSS voltage output under a static load (step response).

To evaluate the sensing stability of the sensors, we simultaneously measured the no loading sensor outputs and step responses of NCSS and the strain gauge under static loads to determine the response performance of the sensors, as shown in Figure 5.

The NCSS voltage output was as stable as that of the foil strain gauge. Under a no loading condition, the maximum absolute deviation of the NCSS voltage output (0.022 mV) was 22% greater than that of the commercial strain gauge (0.018 mV). It was confirmed that very stable voltage outputs were acquired from NCSS through improvements in both the electrical properties of the nanocomposites and signal processing. Generally, it is difficult to obtain such a stable voltage output from the CNT composite sensors due to resistance drift of the composite resistance originating from the inherent electrical changing property of CNTs according to surroundings [24]. However, we succeeded in controlling the resistance stability and obtained stable sensing characteristics from the NCSS. The process and experimental results will be reported in detail later.

**Figure 6:**
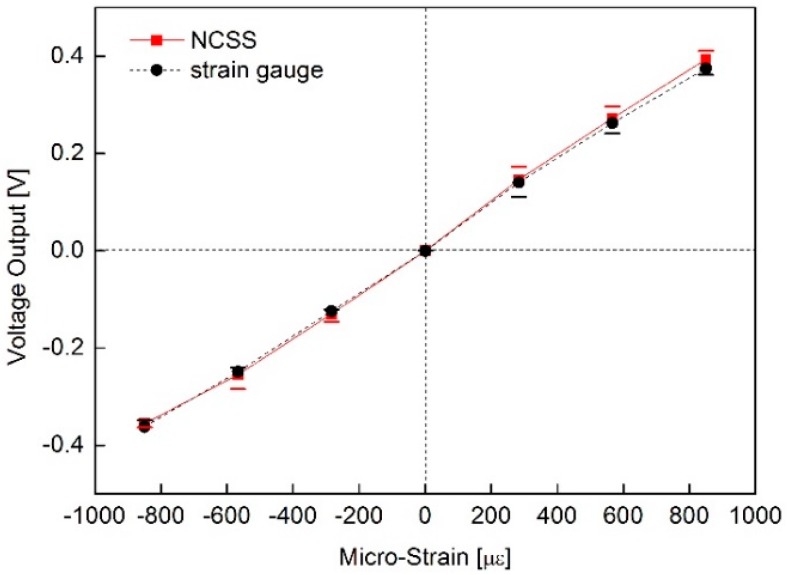
Strain sensing linearity of the MWCNT/epoxy NCSS.

To assess the linearity of the NCSS, the output voltage of the sensor was measured according to the deformation of the cantilever. Figure 6 shows the linear voltage response of the NCSS compared to the strain gauge after signal processing.

In this test, the end point linearity was 3.8% full scale (FS) non-linearity, which was fairly linear for a strain sensor, and the response performances of the two sensors were nearly identical. 

**Figure 7:**
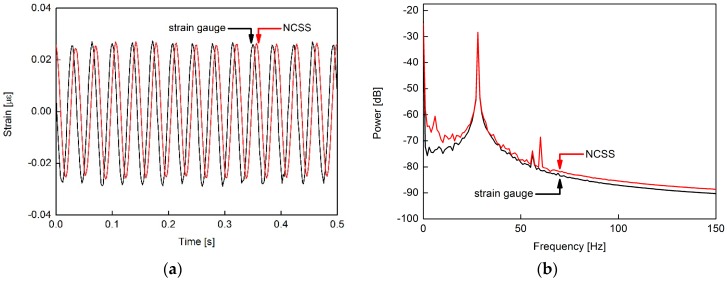
Dynamic characteristics MWCNT/epoxy NCSS; (**a**) voltage output response under 28 Hz excitation and (**b**) NCSS power spectra.

The dynamic characteristics were also tested, as shown in Figure 7.

The NCSS was confirmed for vibration monitoring of the structures. The phase discrepancy between NCSS and the foil strain gauge on Figure 7a should be related to a delay or filtering in the data acquisition. Figure 7b shows the power spectra of NCSS that supports the vibration analysis applications in this study.

The NCSS was validated to measure the reliable static and dynamic strain information under loading and deformed structural conditions. The NCSS made of the MWCNT/epoxy shows linear strain responses compared to those of the foil strain gauge. Thus, it is expected that it can be used as a new strain sensor. It is necessary to investigate the thermal and other characteristics of the NCSS. The MWCNT/epoxy based strain sensor can be fabricated as various types and applied to structural SHM.

## 4. Cross-Sectional Damage Detection in a Vibrating Cantilever

**Figure 8:**
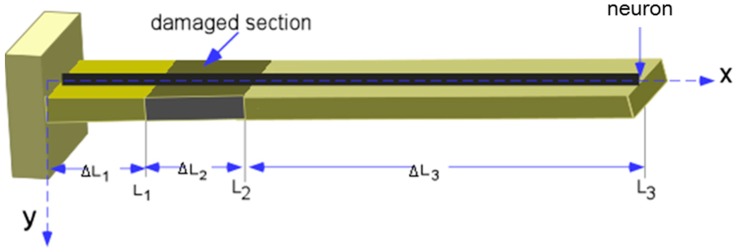
Damaged cantilever beam with a strain neuron.

A strain gauge conventionally measures the point strain, but the neuron can measure the averaged strain along the length of the structure where it was installed. Damages such as corrosion and deterioration can be represented as a change in the cross section of a beam segment. Therefore, this study assumes the beam shown in Figure 8, which has a cross sectional damaged zone wherein the bending rigidity is reduced due to the change in cross sectional area. 

A FE model with ANSYS (Canonsburg, PA, USA) was constructed for the glass fiber cantilever beam to study the dynamic response capacity of the neuron and establish a baseline FE model for subsequent damage assessment based on the vibration response of the neuron. Table 2 outlines the baseline parameters of the beam used in the analysis. The area, moment of inertia of the cross-sectional area, and density in Table 2 were determined from measurements of the beam.

Damage detection of the cantilever beam was performed using the vibration data from the neuron. The local damage, such as a crack, corrosion, or other defect, reduced the local stiffness of the structure that affects the global dynamic characteristics of the structure [21,22]. Therefore, health monitoring may be possible by measuring the change in natural frequencies of the structure. In this study, only the first natural frequency was used because of the canceling effects of some modes (e.g., 3rd mode of the cantilever beam) when measuring the strain along the entire length of the beam, and the first natural frequency is an important parameter to estimate the behavior of the large infrastructure. In addition, it is easier to measure the first frequency than the higher frequencies due to the large amplitude of vibration. 

Element 2 (ΔL_2_) shown in Figure 8 was used as the damaged element because of the relatively large strain induced. The loss of bending rigidity of Element 2 for each damage case was 5.78%, 11.08%, 17.40%, 20.96%, 25.26%, and 30.05%. Six cases of damage were artificially applied by filing both sides of an element to reduce the width of the beam which led a reduction of the beam inertia. The damage ratios were calculated by cross sectional area reductions of the Element 2. 

**Figure 9:**
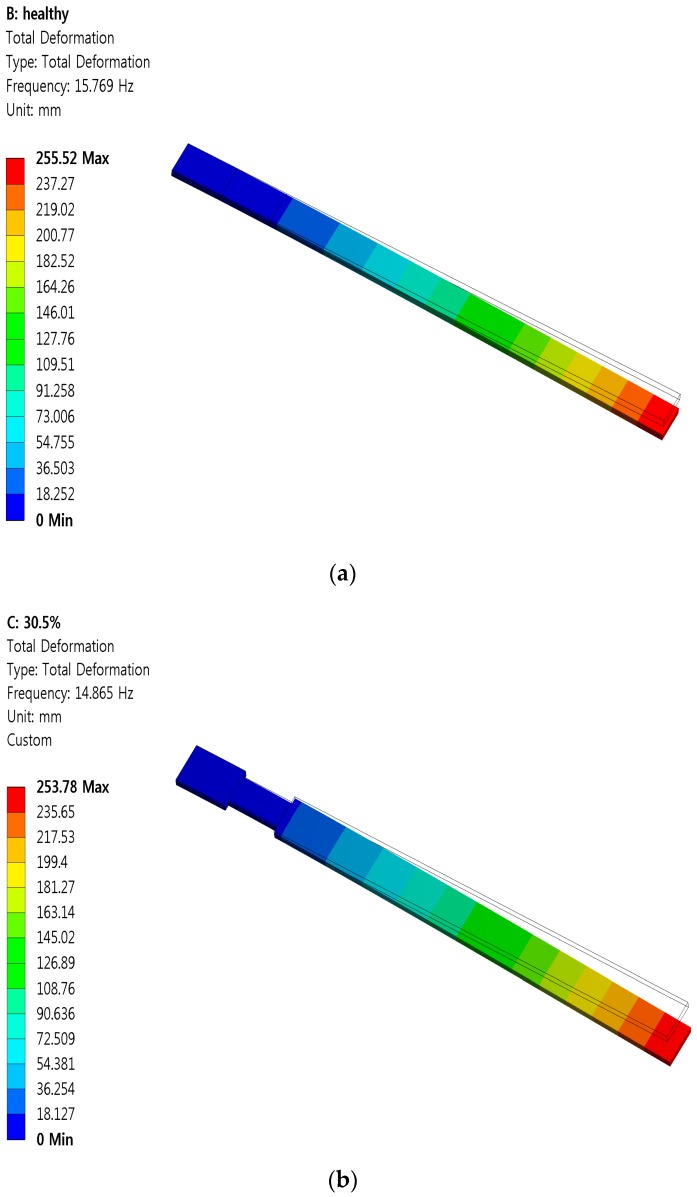
ANSYS analysis examples for the first natural frequencies of the beam; (**a**) healthy beam case and (**b**) 30.5% damaged case.

The first natural frequencies of the beam were analyzed with ANSYS and the results shown in Figure 9.

**Figure 10:**
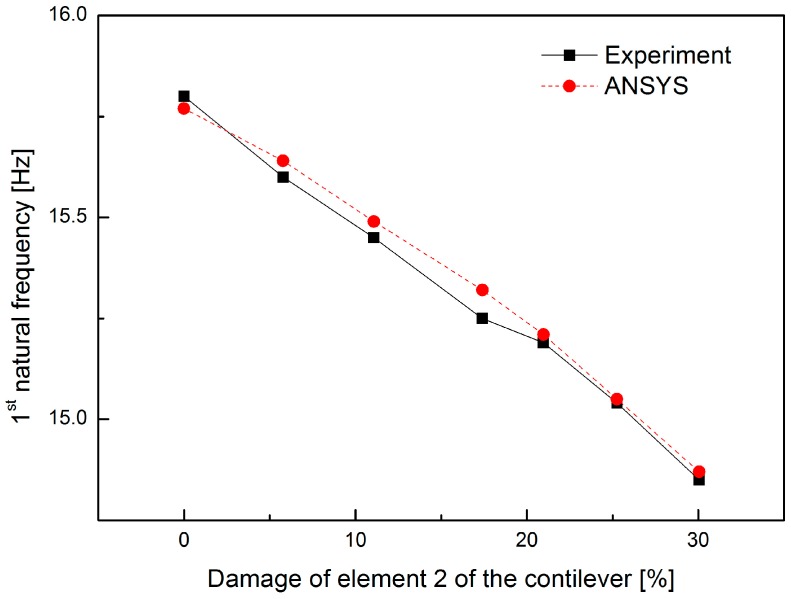
First natural frequencies for various damage cases.

Free vibration tests with the initial displacement method were experimentally performed for a healthy case and the six damage cases. The response of the neuron was sampled at 50 kHz for 5 s. The first natural frequencies for the healthy and damaged beams based on measurements from the vibration tests were compared to those obtained from the ANSYS in Table 3 and Figure 10. The measured vibration response under a structural deterioration closely matched the ANSYS analysis.

## 5. Conclusions

A nanocomposite strain sensor (NCSS) was studied to develop a novel SHM sensor that could be easily installed in a composite structure. The NCSS was fabricated with epoxy and MWCNT as a matrix and nano filler, respectively. The NCSS was patterned on a structure using a spray-on technique, and this made it easy to apply it to existing structures for SHM applications. We accomplished very stable voltage outputs from the NCSS through improvements of both electrical properties of the nanocomposite and signal processing. This study evaluated the NCSS sensing characteristics by benchmarking to those of a conventional foil strain gauge. Under a no loading condition, even though a maximum absolute deviation of the NCSS voltage output was greater than the foil strain gauge, the NCSS voltage output was as stable as that of a commercial sensor. For the linearity test of the NCSS, the end point linearity was 3.8% FS non-linearity, which is quite linear for a strain sensor. The NCSS also showed good performance for vibration monitoring of the structures. The dynamic characteristics of the NCSS voltage output response were nearly identical at the 28 Hz excitation test. The NCSS power spectra proved that it can be available for vibration based NDI techniques. 

The NCSS neuron was developed as a continuous strain sensor. The vibration response of the neuron was investigated for structural damage detection of a composite cantilever. The measured vibration response and damage detection by tracking the first natural frequency showed good results that matched the FE analysis. The spray type, long continuous strain sensor is anticipated to be a versatile sensor in mechanical and civil engineering because of its static and dynamic strain measurement with a longer range and less required data acquisition.

This study confirmed that NCSS might be able to measure reliable information under loading and deformed structural conditions. NCSS can provide assurance of the operational health of a composite structure without the need for actuators or complex wave propagation analysis. The NCSS artificial neuron may effectively save the cost of SHM by reducing the number of channels of data acquisition compared to point sensors covering the same area. Thus, it may be applied to the state diagnosing system for composite structures. It is necessary to perform studies on the investigation of thermal and other characteristics of the NCSS. We plan to present more details on the strain sensing characteristics of NCSS with sophisticated experimental results including environmental conditions.

## Figures and Tables

**Table 1:** Sensor specification.

	Foil Strain Gauge	Nano Composite Strain Sensor (NCSS)
**Resistance [Ω]**	120	2.7 k
**Gauge factor**	2.1	2.8
**Size [mm] w × l × t**	6 × 40 × 0.07	9.15 × 51.76 × 2.85

**Table 2:** Estimated geometric parameters of the beam.

Thickness *t* (m)	Beam width *B* (m)	Estimated E *E* (Gpa)	Density *D* (kg/m^3^)	Length 1 L_1_ (m)	Length 2 L_2_ (m)	Length 3 L_3_ (m)
0.00346	0.024	25.1	1806	0.035	0.035	0.28

**Table 3:** First natural frequencies for various damage cases of Element 2 of the cantilever beam.

	Damage to Element 2 (%)	Experimental (Hz)	ANSYS (Hz)
Healthy	0	15.80	15.77
Case 1	5.78	15.60	15.64
Case 2	11.08	15.45	15.49
Case 3	17.40	15.25	15.32
Case 4	20.96	15.19	15.21
Case 5	25.26	15.04	15.05
Case 6	30.05	14.85	14.87
